# Colour stability of denture teeth: Acrylic versus composite in tea, coffee and turmeric solutions

**DOI:** 10.6026/973206300213157

**Published:** 2025-09-30

**Authors:** Abhishek Chitragar, Mridula Joshi, Mirella Vaz, Nilesha Kadam, Nilesh Joshi

**Affiliations:** 1 Department of Prosthodontics and Crown & Bridge, Bharati Vidyapeeth (Deemed to be University) Dental College and Hospital, Navi Mumbai, India; 2 Department of Periodontology, Bharati Vidyapeeth (Deemed to be University) Dental College and Hospital, Navi Mumbai, India

**Keywords:** Acrylic teeth, color stability, composite teeth, spectrophotometer, staining agents

## Abstract

Color stability of acrylic and composite denture teeth is of interest. Six central incisors from each group were taken and fixed horizontally on the floor of every container, containing tea solution, coffee solution, turmeric solution and distilled water (control group) as the four subgroups and labelled. Solutions were changed every three days and stirred once daily. The spectrophotometer was used for the color measurements of these total 96 teeth before immersion and after one day, one week and four weeks of immersion. The immersion period had a significant impact on the color stability of all the teeth. Composite denture teeth exhibited better resistance to color change as compared to acrylic denture teeth. However, the most chromogenic agent amongst the solutions tested was found to be turmeric.

## Background:

Removable dental prostheses have been used since many years to rehabilitate edentulous patients as it aids in restoring masticatory function, esthetics, self- confidence and capacity for socialization [1, 2]. Denture teeth that are used for the fabrication of complete dentures must have optimum mechanical properties to withstand forces of mastication, should be esthetic and exhibit color stability. Moreover, adequate bond strength to denture base resin is crucial for their longevity. The most commonly used materials for the fabrication of denture teeth are Methacrylate resin, reinforced methacrylate resin, and composite resin. Color stability is a key factor for optimal esthetics of denture teeth [3]. The color stability of denture teeth is one of the most important and clinically relevant optical properties of dental materials since color change indicates aging or deterioration of material. Stain accumulation, water sorption, degradation of intrinsic pigments and increasing surface roughness contribute to color change. The color changes in a removable prosthesis affect its overall esthetics. This might affect patient satisfaction and self-confidence. Removable prosthesis may get exposed to various coloring agents, including certain food or beverages and oral hygiene products, contributing to the pigmentation of the resin matrix. Therefore, color stability of various types of denture teeth after immersion in different beverages and solutions has been investigated extensively [4]. Artificial teeth play a vital role in complete denture and removable partial denture esthetics as well as functional outcomes. Thus, they should possess acceptable color stability [1, 5, 6, 7, 8, 9, 10, 11- 12]. Therefore, it is of interest to evaluate and compare the color stability of acrylic and composite denture teeth after immersion in tea, coffee and turmeric solutions.

## Materials and Methods:

In this study, two brands of acrylic and composite teeth were used. Table 1 (see PDF) shows the brand name, manufacturer, type, shade and mold of the teeth used in this study. Tea, coffee and turmeric were used as staining agent. The details of each staining agent are given in Table 2 (see PDF).

## Preparation of staining solutions [3, 8]:

Preparation of the tea solution was done by immersing five tea bags (Taj Mahal, Brooke Bond) into 1000 ml of boiled water. Coffee solution was the second staining solution used. 20g of coffee powder (Nescafe classic, Nestle, India) was added to 1000ml of boiled distilled water to prepare the coffee solution. The solution was stirred every 30 minutes for 10 seconds until it cools down to room temperature. Then the solution was filtered through a filter paper. Preparation of the turmeric solution was done by adding 20g of turmeric powder to 1000ml boiling distilled water and then cooled for 10 min. It was then filtered through a piece of gauze. Distilled water, the fourth solution, was used as control group.

## Procedure:

Four groups were made - one of tea solution, one of coffee solution, one of turmeric solution and one for distilled water which was used as control. Four containers were hence labelled accordingly. Six central incisors were taken from each group and fixed horizontally on the floor of every container and labelled accordingly. Therefore, each group had four subgroups as per the brand of teeth and comprised of a total of 24 teeth. The four groups accounted to a total of 96 teeth. Before the staining procedure began, all the samples were kept immersed in distilled water for 24 hours. The tea, coffee and turmeric solutions, after it had cooled down to the room temperature, were poured into their respective containers. The distilled water was also poured into their respective container. The containers were then kept in an incubator at 37°C so as to simulate the oral cavity temperature. At three days' interval, the solutions were changed and inorder to minimize the particle precipitate in the solution, it was stirred once daily.

## Color measurements:

The center of the measuring head of the spectrophotometer (X- Rite Pantone Ci7860 benchtop spectrophotometer) was used to hold the specimens, ensuring that it completely covered the aperture of the spectrophotometer. The denture teeth were stored in distilled water at room temperature for 24 hours prior to immersing them into the solutions. Followed by which, the spectrophotometer was used for color measurement of each tooth (T0) after 24 hours of immersion. After recording the first reading (T0), teeth were placed in their respective containers as mentioned above. Following which the subsequent color measurements were recorded after one day (T1), one week (T2), and four weeks (T3) of immersion. Before taking each measurement, the teeth were rinsed in distilled water and the excess water was removed from the surfaces with the help of tissue papers [8]. (Table 3 - see PDF) Using the supplied white calibration standard and in accordance to the manufacturer's instructions, the spectrophotometer was calibrated before taking readings. Measurements were taken from one point which corresponded to the central region of the labial surface of the six teeth. Average value of these six readings were automatically calculated by the spectrophotometer and recorded. The color changes were characterized using the Commission Internationale d'Eclairage L*a*b* color space (CIE L*a*b*). Total color differences are expressed by the formula, ΔE*=[(ΔL)2+ (Δa)2 + (Δb)2 ]1/2, where ΔL, Δa, and Δb are differences in L*, a*, and b* values before (T0) and after immersion at each time interval (T1, T2, T3). The data was converted to National Bureau of Standards units (NBS units) through the equation, (Table 4 - see PDF) NBS units = ΔE* x0.92, where critical remarks of color differences are expressed in terms of NBS units [6] in order to relate the amount of color change (ΔE*) recorded by the spectrophotometer to a clinical environment.

## Results:

The total color difference (ΔE*) and corresponding NBS values of the denture teeth after one day, one week and four weeks of immersion in all the test solutions is shown in Table 5 (see PDF). Results of repeated measures ANOVA indicated that all factors (time, denture teeth, and solution) and all their possible interactions were statistically significant. It was revealed by the Two-way ANOVA that the immersion time significantly affected the color stability of all teeth (p<0.0001) and that the resin material was less resistant to discoloration than the composite material [8]. On evaluating the NBS values at the end of fourth week for all teeth groups, it was observed that turmeric solution caused "much" color difference. On the other hand, only slight color changes were observed in the samples immersed in the other solutions. Moreover, this color change was not noticed by the naked eye. It was also observed that with the increase in the immersion time, the color changes in the denture teeth increased.

## Discussion:

Dental prostheses are in constant contact with oral fluids, food and beverages, and prolonged exposure predisposes them to color changes over time [13]. Color change is an indication of aging or damaging of the materials. This makes color stability one of the most important clinical properties for denture teeth. Furthermore, the esthetic appearance of prosthesis must satisfy the patient's expectations and is certainly considered as an important feature [8, 14 and 15]. The color alterations of dental materials can be assessed either visually or using instrumental techniques. Studies indicate that instrumental evaluation offers superior accuracy in detecting minimal color changes on flat surfaces [16]. The use of instrumental technique eliminates the subjective interpretation of visual comparison, and hence is the reason behind the wide use of spectrophotometers. Color changes are characterized using the Commission Internationale d'Eclairage L*a*b* color space. This color space is one of the most popular and widely used color spaces and is well suited for the determination of small color differences [8, 14 and 15]. The CIE Lab system is a uniform three-dimensional model used to evaluate color changes. It is widely applied for assessing chromatic differences and offers advantages over the Munsell color system, as instrumental measurement minimizes the subjective bias associated with visual color comparison. In this system, the three dimensions of color are arranged at nearly equal intervals, and the evaluation of color differences-derived from various combinations within the three-dimensional color space-provides objective mathematical data. For this study, the CIE Lab system was employed, and spectrophotometric techniques were used to measure the color of three types of acrylic resin in three different test media [17]. In this three - dimensional color space, the three axes are namely L*, a* and b*. The L* value is a measure of the whiteness or brightness of an object. The a* value is a measure of redness (positive a*) or greenness (negative a*). The b* value is a measure of yellowness (positive b*) or blueness (negative b*). The advantage of this system is that color differences can be expressed in units that can be related to visual perception and clinical significance [8, 14 and 15]. The formula to calculate ΔE* has been mentioned previously. Many authors have used ΔE* values to evaluate the "perceptibility" of color differences. Since the criteria of perceptibility adopted varied between the authors, the NBS Rating System was employed so as to counter the differences and disagreements hence caused [18, 19, 20, 21, 22-23].

The NBS Rating System is a frequently used method to determine the degree of color difference since it offers absolute criteria by which ΔE* values can be converted to remarks with clinical significance [16]. Ehsani *et al.* conducted a study to assess the color change (ΔE00) of 7 brands of denture teeth (conventional acrylic and composite teeth) namely; Finex, Emeral, Phonares II, Vitapan, SR Vivodent PE, Crystal and Beta Star following immersion in staining solutions such as tea, coffee, cola and turmeric. It was observed that after 1-month immersion of denture teeth in the staining solutions, there was an alteration in the ΔE values; however, they were within the acceptable range, except for Beta Star. It was seen that turmeric solution caused unacceptable color change in all denture teeth even after 24 hour of immersion [3]. Bitencourt *et al.* conducted a study to evaluate the effect of acidic beverages on surface roughness and color stability of artificial teeth and acrylic resin. In general, the acidic solutions changed the roughness and color change (ΔEab) of heat polymerized acrylic resin and artificial teeth after immersion in acidic solution for 10mins for 14 days. The grape juice altered the roughness only of the artificial teeth, promoting a clinically acceptable color change in the materials [24]. Tieh *et al.* conducted a systematic review on the Optical properties and color stability of denture teeth. They concluded that the color stability of CAD/CAM milled denture teeth is comparable to conventional PMMA denture teeth. There are contradictory findings in terms of color stability of 3D printed denture teeth as compared to conventional PMMA denture teeth. It was found that staining by coffee is worst among the common beverages and solutions investigated. Denture teeth can show color changes after immersion in staining beverages as early as one week. The degree of discoloration of denture teeth after immersion is time dependent, with the larger extent in the initial phase [4]. Dimitrova *et al.* conducted an *in-vitro* study to evaluate the color stability of 3D- printed and prefabricated denture teeth after immersion in different coloring agents such as coffee, red wine and coca-cola. It was seen that all the specimens demonstrated an increased color change after 7 days compared to after 21 days and the difference in mean was statistically significant [25]. In the present study, therefore, corresponding NBS values were calculated to assess the color differences caused by immersion time and / or solution type. It was found that the NBS values of all teeth groups immersed in turmeric solution were greater than 6, meaning that color change was "much". Earlier studies have confirmed that lighter materials discolor more prominently than darker materials. Thus, denture teeth of shade A1 were selected for this study because they exhibit staining solutions more distinctly [8, 26]. In the present study, acrylic denture teeth were compared against composite denture teeth. It was found that composite denture teeth exhibited less color change as compared to acrylic denture teeth in all the three staining solutions. Among the staining solutions used, turmeric solution was the most chromogenic solution. When NBS values at the fourth week of immersion period were evaluated, "much" color changes were observed in all teeth groups immersed in turmeric solution. Color changes in tea and coffee solutions were only "slight changes" according to the NBS System. Color differences with corresponding ΔE* values lower than 3.3 are acceptable in clinical dentistry [20, 26]. In the current study, ΔE* values of all groups remained in the clinically acceptable range except for the groups immersed in turmeric solution.

## Limitations of the study:

Though the study was designed and carried out with utmost accuracy, it has certain limitations that, in spite of following a standard protocol for the preparation of staining solutions, the degree and homogeneity of the filtration done could not be controlled. Also, thermocyling was not done in the study.

## Scope for further studies:

The present study focused on the color stability of acrylic and composite denture teeth. Further studies on other factors like wear resistance, darker shades of teeth, bonding to denture bases, effect of thermocycling and various other mechanical properties could be conducted. The addition of more types of denture teeth such as porcelain denture teeth and reinforced acrylic denture teeth can be helpful. The staining solutions and time intervals can also be altered. These parameters should also be considered so as to boost the external validity of this study.

## Conclusion:

Acryrock is more color stable as compared to Cosmo HXL when subjected to staining solutions, at different time intervals for acrylic teeth. Veracia SA is more color stable as compared to SR Phonares II when subjected to staining solutions, at different time intervals for composite teeth. Composite teeth exhibited more color stability as compared to acrylic teeth, when subjected to immersion in staining solutions for four weeks. Veracia SA displayed most color stability when compared with all brands of teeth used in the study. Turmeric solution turned out to be the most chromogenic staining solution as compared to tea and coffee solutions.
